# Advancing universal health coverage through non-medical interventions: the evolution and equity of China’s National Fitness policy system

**DOI:** 10.3389/fpubh.2026.1770159

**Published:** 2026-02-20

**Authors:** ZhiHua Wang, Pingping Zhou, Chuan Mou

**Affiliations:** 1 College of Physical Education, Sichuan University, Chengdu, China; 2 Paotongshu Primary School, Chengdu, China

**Keywords:** LDA, National Fitness, non-medical interventions, policy tools, social network analysis

## Abstract

**Background:**

Physical inactivity is a primary driver of non-communicable diseases (NCDs), posing a significant threat to the fiscal and operational sustainability of Universal Health Coverage (UHC). In China, the National Fitness initiative serves as a critical preventive health framework within the “Healthy China 2030” strategy. This study evaluates China’s 70-year evolution of physical activity policies as a large-scale, non-medical public health intervention, aiming to provide governance lessons for global health systems seeking sustainable UHC solutions.

**Methods:**

We analyzed 145 central-level policy documents issued between 1949 and 2021 using a mixed-methods approach. Specifically, We employed quantitative attribute assessment for temporal mapping, Social Network Analysis (SNA) to identify interdepartmental governance structures, Latent Dirichlet Allocation (LDA) for thematic priority modeling, and qualitative coding based on Ramesh’s framework to evaluate the distribution of policy instruments for health promotion.

**Results:**

Policy activity has surged since 2012 (74% of current policies), reflecting a strategic shift toward preventive healthcare. The governance network exhibits a centralized structure where the General Administration of Sport collaborates with a “Golden Triangle” of the Ministries of Education, Health, and Finance, though broader multisectoral integration remains nascent. Thematic analysis reveals a focus on “hardware” foundations (facilities, funding) and vulnerable populations (youth and older adults), aligning with UHC’s equity goals. However, gaps persist in “sports–health integration” and digital health adoption. Policy instruments are heavily skewed toward command-and-control measures (68.4%), with market-based and voluntary incentives significantly underutilized.

**Conclusion:**

China’s model demonstrates how state-led, non-medical interventions can rapidly expand preventive service coverage. To ensure long-term UHC sustainability, the governance model must transition from an infrastructure-centric, state-led approach to a multi-stakeholder, integrated governance framework. Enhancing interdepartmental coordination (“Fitness in All Policies”) and rebalancing policy instruments are essential for building a resilient, equity-oriented public health system that reduces the long-term burden on curative medical services.

## 1 Introduction

Health is not only central to individual well-being, but also a fundamental driver of social and economic development (1). Achieving health is not only an ultimate goal, but also an effective means of achieving a range of broader social goals, such as population well-being, economic development, environmental sustainability, and social cohesion (2), while significantly alleviating the economic burden of disease (3). Therefore, improving the health of the population has become a core agenda item for governments around the world and is occupying an increasingly prominent position in global health policy (4). However, there is growing recognition that health is significantly influenced by factors beyond the scope of the health sector (5). Relying solely on traditional medical models centered on disease treatment not only poses unsustainable fiscal challenges but also fails to achieve fundamental improvements in population health (6). In this context, non-medical interventions, particularly the promotion of regular physical activity, have been widely proven to be an efficient and cost-effective strategy for effectively curbing the continued spread of non-communicable diseases such as cardiovascular disease and diabetes (7). This has led to the advocacy of physical exercise becoming an indispensable part of public health measures (8). However, there remains a critical gap in global health policy research: despite the recognition of the value of physical activity, the governance mechanisms required for implementing such interventions at the national level, particularly in the context of developing countries, have not been sufficiently explored.

China’s National Fitness policy provides a unique case, demonstrating how to address the complex challenges of public health governance. As one of the largest national initiatives in the world, it aims to use sports and physical activity as non-medical means to systematically improve public health. National Fitness is not only the cornerstone of the grand vision of “Healthy China 2030,” but has also been explicitly incorporated into national top-level design, aiming to establish a comprehensive health system for 1.4 billion citizens (9). From the evolution of China’s National Fitness policy, its institutionalized origins can be traced back to the promulgation of the “Outline of the National Fitness Program” in 1995, a landmark document marking the government’s systematic promotion of public sports participation (10). The successful hosting of the 2008 Beijing Olympics injected strong momentum into this effort, shifting the national sports focus toward the popularization of mass sports (11). Subsequently, the implementation of the “Regulations on National Fitness” in 2009 and the issuance of a series of documents such as the “National FitnessPlan (2021–2025)” not only consolidated the legal status of National Fitnessbut also deepened its integration with the nation’s long-term public health and development strategies (12). However, despite the ambitious policy goals, it remains unclear whether the existing policy tools and collaborative networks can effectively support this strategic transformation, particularly in terms of whether they structurally align with the nation’s grand vision.

Despite the significant importance of China’s National Fitness policy, existing research mainly focuses on the effects of specific interventions or provides qualitative historical overviews, lacking a systematic evaluation of the internal logic of policy design. To fill this gap and provide transferable insights for Universal Health Coverage (UHC), this study goes beyond descriptive analysis by empirically evaluating “National Fitness” as a key large-scale “non-medical intervention” for maintaining the sustainability of UHC. The study raises a core research question: How can the governance logic of China’s National Fitness policy effectively evolve to support the achievement of public health goals? At the same time, can the existing policy tools effectively support this strategic transformation?

To accurately deconstruct this complex policy system, this study constructs a comprehensive text analysis framework. We adopt a multi-dimensional theoretical perspective to systematically examine the structure of China’s National Fitness from the following four interrelated dimensions: (1) Policy text attributes, analyzing the quantity, types, and temporal distribution patterns of policy documents; (2) Policy actors, identifying the core institutions responsible for policy formulation and their collaborative network patterns; (3) Policy themes, using computational methods to objectively identify the core issues addressed by the policies; (4) Policy tools, categorizing and analyzing the specific governance measures adopted by the state to achieve its strategic objectives. Through this framework, this study aims to make the following key contributions: First, it presents the first data-driven panoramic map of this critical public health strategy, transcending existing descriptive analyses. Second, it reveals the intrinsic structural characteristics and potential imbalances of the National Fitness policy supply system, providing empirical insights into the logic of state-led public health governance in China. Finally, this study will provide a robust evidence base for policymakers to optimize National Fitness strategies, thereby contributing valuable knowledge and experience to the design and implementation of large-scale non-medical health interventions globally.

## 2 Materials and methods

### 2.1 Data collection and corpus construction

The policy documents on National Fitness refer to various codes of conduct formulated by relevant government departments to meet the public’s fitness needs, correct abnormal phenomena in the development of National Fitness, and complement national strategic objectives. These codes include laws and regulations formulated by legislative bodies, as well as general policy documents such as opinions, measures, notices, and regulations issued by administrative agencies and the ruling party. The essence of the policy documents on National Fitness is the integration and allocation of national public sports resources (13).

Establishing a comprehensive and representative corpus of policy documents forms the foundation of this study. Data collection employed two primary methods. First, the Directory of Current Valid Sports Laws, Regulations, Rules, Normative Documents, and Institutional Documents (as of December 31, 2021), published by the National Sports Administration, was used as a source, with each policy manually reviewed and collected. Second, the Peking University Law Database,^1^ China’s leading legal information platform, was searched in full text using the keyword “National Fitness.” The results from both sources were cross-referenced to determine the final sample. To ensure representativeness, the following principles guided the screening and organization of texts:

(1) Public accessibility. Policies must be publicly available through government portal websites, mainstream media reports, sports yearbooks, or other open channels. Non-public documents were excluded.
(2) Authority. Only policies issued by central-level institutions (e.g., the National People’s Congress, the State Council, ministries, commissions, and their affiliated agencies) were included, given their overarching guiding significance. Local policies and regulations were excluded.
(3) Uniqueness. Duplications caused by variations in naming conventions (e.g., Notice of the General Office of the State Council Forwarding the Opinions on Strengthening the Management of Fitness Qigong Activities by the General Administration of Sport, the Ministry of Civil Affairs, and the Ministry of Public Security versus Opinions on Strengthening the Management of Fitness Qigong Activities) were resolved by retaining the version issued by the original authority. Notices containing multiple policies (e.g., Notice from the General Office of the General Administration of Sport on the Implementation of the “Technical Specifications for the Information Management and Service System of Sports Venues” and the “Data Interface Specifications for the National Fitness Information Service Platform”) were treated as individual policies and kept under their original titles.
(4) Timeliness. Only policy documents issued between 1949 and 2021 that remain in effect were considered. For documents with multiple revisions, amendments, or corrections, the latest version was adopted.
(5) Relevance. Policies had to directly concern the development of National Fitness, containing substantive content or explicit measures for its promotion. Documents that merely mention National Fitness without concrete measures were excluded, as were those with limited practical significance (e.g., replies, letters, or meeting notices).

After collecting, screening, and cross-validating the data, a preliminary database of National Fitness policies was established. Subsequently, five experts in the field of sports policy were invited to refine the corpus to ensure its quality (expert information is provided in the Supplementary materials 1). Finally, 145 National Fitness policy documents were selected for analysis (a partial list is provided in Table 1, and the complete list can be found in the Supplementary materials 2).

**Table 1:** Data table of China’s National Fitness policy documents (1949–2021) (partial).

Serial no	Policy name
1	Notice from the Ministry of Urban and Rural Development and Environmental Protection and the State Sports Commission on the Interim Provisions on the Quota Standards for Urban Public Sports Facilities Land Use
2	Management Measures for Powered Paragliding Activities
3	Notice from the General Administration of Sport of China, the Ministry of Civil Affairs, and the Ministry of Public Security on Opinions Regarding the Strengthening of Management of Fitness Qigong Activities
……	……
143	Notice from the General Administration of Sport on the Issuance of the “Opinions on Strengthening the Management of Public Fitness Equipment in Public Places”
144	Notice from the General Administration of Sport on the Issuance of the “Basic Public Service Standards for Mass Fitness (2021 Edition)”
145	Outline of the 14th Five-Year Plan for National Economic and Social Development of the People’s Republic of China and the Long-Term Objectives Through the Year 2035

### 2.2 Analytical techniques

#### 2.2.1 Policy text attribute analysis

To better understand the distribution of China’s National Fitness policies, this paper introduces a policy intensity indicator and, drawing on the approaches of Peng et al. (14) and He et al. (15), establishes a quantitative standard for the intensity of China’s National Fitness policies (Table 2). This standard is used to quantify and measure political priority levels, enabling a longitudinal analysis of government attention.

**Table 2:** Intensity of National Fitness policies and scoring criteria.

Policy issuing authority	Scoring criteria
Laws, regulations, and planning guidelines issued by the National People’s Congress and its Standing Committee	5
Regulations, rules, plans, programs, and guidelines issued by the Central Committee of the Communist Party of China and the State Council	4
Interim regulations, interim rules, opinions, measures, plans, and standards issued by the Central Committee of the Communist Party of China and the State Council; regulations, rules, plans, and programs issued by various ministries and commissions	3
Interim regulations, interim rules, opinions, measures, plans, standards, and norms issued by various ministries and commissions	2
Notices issued by the State Council and various ministries and commissions	1

#### 2.2.2 Social Network Analysis (SNA)

To illustrate the cross-departmental governance structure, we focused on 60 documents jointly issued by two or more government agencies and statistically analyzed the collaborative relationships between policy entities. In terms of relationship assignment, if two policy entities have jointly issued documents, the relationship between them is assigned a value of 1; otherwise, it is assigned a value of 0. Based on this, the joint issuance matrix for the National Fitness policy was obtained. Subsequently, the UCINET6.560 software was used to calculate the cooperation network structure indicators, and the Netdraw software was used to draw the cooperation network relationship map of Chinese policy entities issuing documents. Three key indicators were mainly analyzed:

(1) Network density. Measures the overall connectivity of the network, reflecting the level of collaboration between departments.
(2) Degree centrality. The number of direct connections to a node, identifying the most active core institutions.
(3) Intermediary centrality. Measures the extent to which a node is located on the shortest path between other nodes, identifying the “bridge” roles connecting different parts of the network.

#### 2.2.3 LDA topic modeling

**Figure 1:**
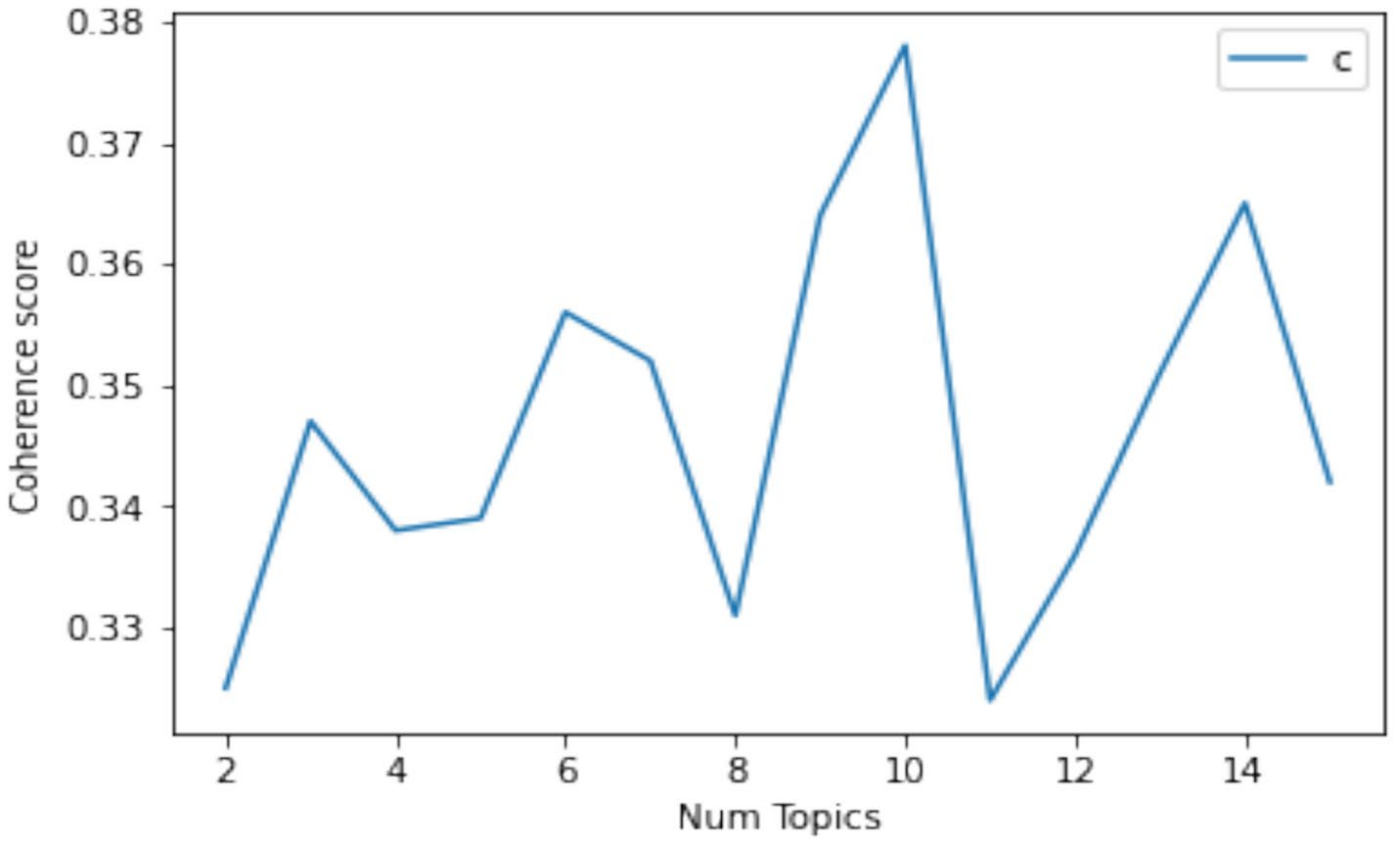
Theme consistency score.

By constructing a topic model of National Fitness policy texts using LDA, we can extract the core essence of the policy and accurately grasp the connotation of China’s current National Fitness policy. The research process consists of the following four parts: (1) Select the 145 policy documents screened and sorted in the above text as the corpus for analysis. (2) Preprocessing of policy text data. The text was segmented and stop words were removed. Specifically, the jieba Chinese word segmentation library in Python was used to segment the policy text content and remove stop words. During this process, stop word lists, word segmentation lists, and synonym lists were added to obtain optimal word segmentation results. (3) Policy text topic generation. The LDA model toolkit was used to model the topics of the National Fitness policy, and the number of topics was determined by calculating the topic consistency score to ensure the interpretability of the model. (4) Policy text topic visualization and analysis. LDA is a topic generation model based on a three-layer Bayesian probability model, including three layers of structure: words, topics, and documents (16). Based on the research results, the pyLDAvis package was used to visualize themes—keywords and conduct corresponding analysis. When modeling the LDA theme model, three calculation methods were provided to determine the optimal number of themes: theme confusion score, theme similarity score, and theme consistency score. Among these, the theme consistency score is more effective in assessing the quality of the model (17, 18). Therefore, this paper calculates the theme consistency score to determine the optimal number of policy themes. According to the implications of the theme consistency score, the higher the consistency score, the better the text clustering effect. As shown in Figure 1, when the number of themes in the National Fitnesspolicy text is 10, the consistency score is the highest. Therefore, we set the number of themes (K) to 10. The research team qualitatively labeled each theme based on high-frequency keywords and representative document interpretations.

#### 2.2.4 Policy tool coding

Using MAXQDA Analytics Pro 2022 qualitative analysis software, the policy content was coded and statistically analyzed based on Ramesh’s (19)-category framework. First, a detailed coding scheme was developed to ensure reliability (Table 3), and each policy provision in the Chinese National Fitness-related policy texts was coded with specific tool types. Second, for policy texts focused on specific areas such as venue regulations and standard implementation, where policy tools are limited, the entire policy text was treated as a single code. For comprehensive National Fitness policy texts, key themes such as core tasks and organizational guarantees were selected for coding (20). Finally, to ensure objectivity and accuracy in coding results, this study employed repeated coding, i.e., coding the policy texts in forward and reverse order at different time points until the coding results stabilized. When a single policy provision involves multiple codes, multiple discussions are conducted to apply multiple codes. Some analytical units are detailed down to specific provisions within a clause, meaning that relevant clauses are split based on specific circumstances to add sub-codes at the next level, ensuring the completeness of the coding of policy documents. During coding, to enhance the accuracy of analytical results, following existing practices, the research background section of the text is not considered.

**Table 3:** Policy tool types.

Tool type	Tool name	Specific content
Voluntary tools	Voluntary organizations	Volunteer organizations, sports social organizations, grassroots cultural and sports organizations
	Family and community	Family mutual aid, family gatherings, community activities
	Market	Supply and demand mechanisms, competition mechanisms
Mandatory tools	Regulation	Laws, systems, rules, standards, supervision, licenses, penalties
	Direct provision	Direct production, direct services, direct management, government procurement
	Command and authority tools	Command execution, institutional adjustments and reforms, government agreements, guidance, plans
Hybrid tools	Information and persuasion	Information disclosure and publication, encouragement and calls to action, appeals, symbols, persuasion
	Subsidies	Grants, rewards, interest rate incentives, tax incentives
	Contracts	Service outsourcing, public-private partnerships
	Incentive tools	Social reputation, trust, decentralization of power, procedural simplification

## 3 Results and analysis

### 3.1 Policy text attributes

#### 3.1.1 Distribution of policy text quantity

**Figure 2:**
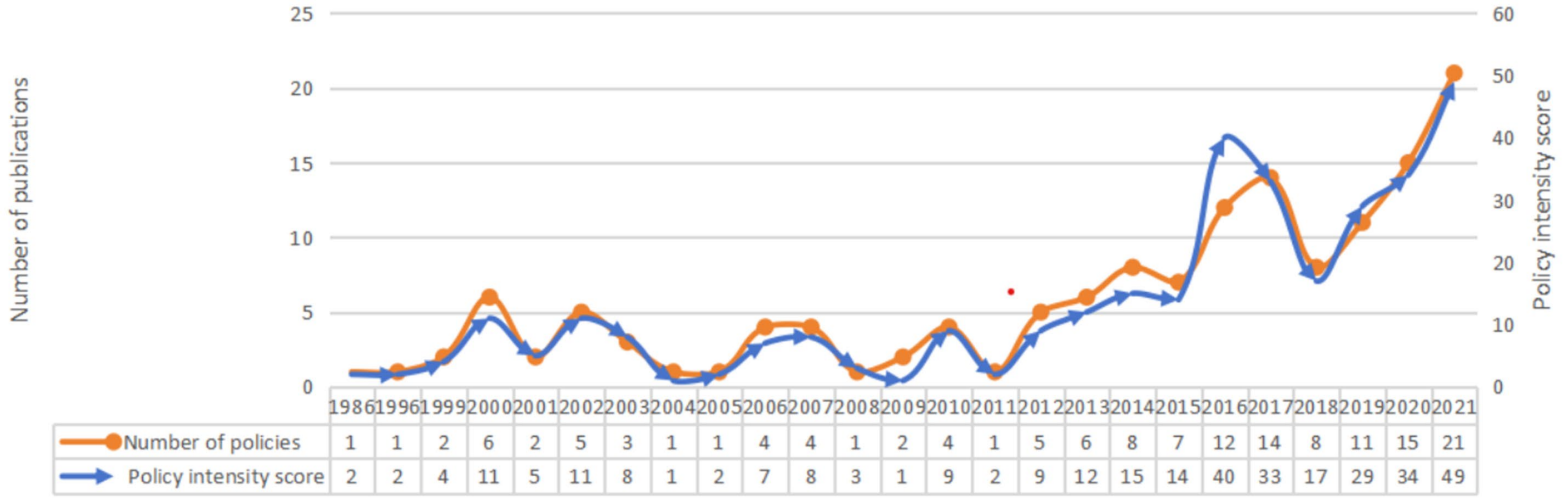
Annual changes in the number of China’s National Fitness policies in China and policy effectiveness scores.

First, while the overall National Fitness policy demonstrates a certain degree of timeliness, there remains room for improvement. As shown in Figure 2, since the 18th National Congress of the Communist Party of China (2012), a total of 107 National Fitness policies have been issued, accounting for 74% of the current valid policies. This makes the policy system relatively timely, reflecting the country’s recent new deployments and requirements in the field of National Fitness. However, some of the existing policy measures are relatively outdated and no longer align with the latest guiding principles, concepts, or the current socio-political context, resulting in low timeliness and hindering their role in promoting National Fitness development. For example, the “Interim Provisions on the Normative Indexes for Public Sports Facility Land Use in Urban Areas,” issued by the Ministry of Urban and Rural Construction and the State Sports Commission in 1986, has not been revised for 36 years. Given the reforms in government institutions, administrative methods, and regulatory standards, the legal and administrative effectiveness of this document has become increasingly unclear, making it difficult to adapt to the current national situation (21). Additionally, the “Management Measures for National Sports Venue Maintenance Special Subsidy Fund,” implemented in 1999 to strengthen the management of maintenance funds for sports venues, states that one of its goals is to promote the “National Fitness Plan.” However, it restricts the scope of funding to the maintenance and renovation of sports venues and equipment for Olympic key projects and sports training bases, which has limited the policy’s impact on the maintenance of public sports facilities. Over time, these policies have lost their effectiveness in promoting National Fitness and may further exacerbate existing problems in the field.

**Figure 3:**
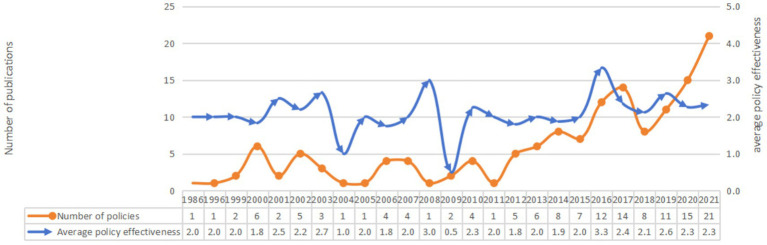
Changes in the number of China’s National Fitness policies in China and average policy effectiveness.

Second, the intensity of National Fitness policies exhibits distinct stage-specific characteristics. Policies issued prior to 2012 remain in effect to a limited extent, with a total of 38 such policies accounting for 26% of all currently effective policies. Since the 18th National Congress of the Communist Party of China, the intensity of National Fitness policies has significantly increased, with the average effectiveness of policies remaining relatively stable (Figure 3). The continuity of policy intensity is strong, with a total of 34 policies at the State Council level or above, 29 of which were issued since 2012, accounting for 85% of the total. Among these, there was 1 policy in 2013, 1 in 2014, 2 in 2015, 7 in 2016, 3 in 2017, 2 in 2018, 5 in 2019, 4 in 2020, and 4 in 2021. The concentrated distribution of current effective policies clearly demonstrates the significant increase in the national government’s attention to physical fitness in recent years.

#### 3.1.2 Distribution of policy text categories

As shown in Table 4, China’s National Fitness policies encompass a diverse range of policy document types, including opinions, measures, notices, plans/outlines/guidelines/programs, regulations, ordinances, standards, schemes, laws, and provisions, totaling ten distinct forms. By analyzing and summarizing these policy document types, the following characteristics of China’s National Fitness policy documents can be identified:

**Table 4:** Statistical table on the composition of policy document types for China’s National Fitness policy.

Document type	Measure	Law	Program	Provision	Plans (plans, outline plans, outlines, programs)	Norm	Regulation	Standard	Notice	Opinion
Document quantity	25	3	8	4	15	4	3	2	20	61
Proportion	17.2%	2.1%	5.5%	2.8%	10.3%	2.8%	2.1%	1.3%	13.8%	42.1%

### 3.2 Policy text structure

#### 3.2.1 Composition of policy issuing entities

Understanding the composition of policy-making entities enables us to identify which government agencies and departments are involved in the development of National Fitness initiatives, addressing the critical question of “who formulates policies.” It also provides insight into the current authority of National Fitness policies and the extent to which they exert control and regulation over individual or group behavior. Based on the number of entities involved in policy formulation, policy documents can be categorized into two types: those issued by a single policy-making entity and those issued by multiple policy-making entities. In documents issued by multiple policy-making entities, this paper determines the primary issuing entity based on the document number and the order of issuing entities listed in the policy document. Additionally, for policy-issuing entities that have been dissolved, merged, or renamed, the latest (2018) government institutional names are used for counting purposes. For example, “Ministry of Health” and “National Health and Family Planning Commission” are both counted as “National Health Commission,” and “Ministry of Culture” and “Ministry of Tourism” are uniformly recorded as “Ministry of Culture and Tourism.” In years where both appear, they are counted only once. The statistical results are shown in Table 5.

**Table 5:** Statistics on the composition of policy-issuing bodies in China’s National Fitness policies.

Policy-issuing agency	Independent issuance	Joint issuance	Total
General Administration of Sport of China (State Physical Culture and Sports Commission, General Office of GASC)	56	55	111
State Council (General Office of the State Council)	25	5	30
National People’s Congress (Standing Committee of the NPC)	4	0	4
Ministry of Education (General Office of MOE)	0	21	21
National Health Commission (Ministry of Health, National Health and Family Planning Commission, General Office of NHC, General Office of State Administration of Traditional Chinese Medicine)	0	16	16
Ministry of Civil Affairs (General Office of MOCA), Ministry of Finance (General Office of MOF)	0	15	15
National Development and Reform Commission (State Planning Commission)	0	14	14
Ministry of Culture and Tourism (China National Tourism Administration, Ministry of Culture)	0	12	12
All-China Federation of Trade Unions, Ministry of Housing and Urban–Rural Development (Ministry of Urban and Rural Construction and Environmental Protection, Ministry of Construction)	0	10	10
Ministry of Public Security, State Administration for Market Regulation (State Administration for Industry and Commerce, SAMR, General Office of SAMR), Communist Youth League Central Committee	0	9	9
All-China Women’s Federation (General Office of ACWF)	0	8	8
Ministry of Agriculture and Rural Affairs, Ministry of Natural Resources (Ministry of Land and Resources)	0	7	7
Ministry of Human Resources and Social Security (Ministry of Labor and Social Security, Ministry of Personnel, MOLSS, General Office of MOHRSS)	0	6	6
State Ethnic Affairs Commission, Central Committee of CPC (General Office), National Radio and Television Administration (State Administration of Press, Publication, Radio, Film and Television; General Administration of Press and Publication; SARFT; China Media Group)	0	5	5
China Disabled Persons’ Federation	0	4	4
Central Civilization Office, Ministry of Emergency Management, Ministry of Industry and Information Technology	0	3	3
Ministry of Science and Technology (General Office of MOST), Publicity Department of CPC Central Committee (General Office), National Committee on Ageing, State Taxation Administration, China Banking and Insurance Regulatory Commission, National Forestry and Grassland Administration, All-China Sports Federation (Inter-ministerial Joint Meeting Office for Football Reform and Development of the State Council / Chinese Football Association)	0	2	2
Ministry of Justice, General Office of Chinese Academy of Sciences, Organization Department of CPC Central Committee, National Bureau of Statistics, State Commission Office for Public Sector Reform, Ministry of Transport, China Meteorological Administration, National Healthcare Security Administration (General Office), Political Department of PLA, Agricultural Development Bank of China, People’s Bank of China, Work Committee of Central and State Organs, National Cultural Heritage Administration, Ministry of Water Resources, Ministry of Foreign Affairs	0	1	1

(1) The “diversity and relative weakness” of policy document efficacy. On one hand, policy texts encompass a range of legal and administrative regulations, ensuring the scientific nature of National Fitness promotion while also facilitating the continuity and connection between policies. On the other hand, As of the end of 2021, there are three laws closely related to National Fitness in China: the Sports Law of the People’s Republic of China (2016 revision), the Basic Medical and Health Promotion Law of the People’s Republic of China, and the Public Cultural Services Guarantee Law of the People’s Republic of China. The majority of the remaining texts are normative policy documents, which clearly lack strong enforceability in promoting National Fitness. It is worth noting that these laws also have certain shortcomings in ensuring the foundational role of National Fitness. In the Sports Law, the term “National Fitness” is mentioned only twice, while the concept of “social sports” is more commonly used. However, “social sports” is typically defined as the category outside of school sports and competitive sports, and its scope is unable to encompass the all-inclusive, all-cycle, and all-society attributes required for “National Fitness” as a national strategy. This conceptual limitation has led to the failure of the law to fully reflect the grand scale and central role of the National Fitness strategy. In the Basic Medical and Health Promotion Law, National Fitness is only a small part of health promotion, limiting its legislative impact. Furthermore, since China currently lacks a dedicated National Fitness Law, the stability and consistency of National Fitness policies are relatively weak, with frequent changes. Therefore, increasing the number of legally effective policy texts is a crucial step in improving the National Fitness policy framework.
(2) Policies are highly normative and directive but lack operational feasibility. As of the end of 2021, China’s National Fitness policies are primarily issued in highly normative and directive policy documents, such as laws, notices, opinions, plans, regulations, and rules, totaling 106 policy documents, accounting for 73% of the total. In contrast, policy documents with higher operational feasibility, such as methods, standards, norms, and plans, total 39, accounting for 27% of the overall policy sample. This indicates that most policies formulated by the central government primarily serve a macro-planning and guiding role. To ensure effective implementation of these policies, local governments need to further develop specific, actionable policy measures tailored to their local circumstances. This highlights that there is significant room for expansion in the types of policy documents with stronger operational feasibility within China’s National Fitness policies.
(3) Policies exhibit a distribution pattern characterized by “strong regulatory intensity and high subject-relatedness.” According to existing research, regulatory intensity and subject-relatedness can serve as criteria for classifying policy documents. In terms of regulatory intensity, legal documents such as laws, regulations, rules, and notices all use language that is “command-oriented,” indicating the strongest regulatory intensity, and are classified as the first tier. Other policy document types such as opinions, plans, and measures have relatively weaker regulatory intensity and are classified as the second tier (22). In terms of subject-relatedness, notices and opinions have clear targeting and hierarchical relationships in their issuing entities, resulting in the highest subject-relatedness, placing them in the first tier of subject-relatedness. Laws, regulations, plans, rules, and methods lack clear targeting in their issuing entities, resulting in lower subject-relatedness, placing them in the second tier of subject-relatedness (23). Based on this statistical analysis, the total number of policy documents with higher control levels is 84, accounting for 58% of the total volume of National Fitness policy documents; the total number of policy documents with higher subject relevance is 121, accounting for 83%. Therefore, China’s National Fitness policies exhibit a distribution pattern characterized by strong control and high subject relevance, indicating strong applicability of the policies.

As of the end of 2021, Statistics show that there are 47 government agencies involved in the formulation of National Fitness policies in China, including the General Administration of Sport of China, the State Council, the Ministry of Education, the National Health Commission, the Ministry of Civil Affairs, the Ministry of Finance, the National Development and Reform Commission, the Ministry of Culture and Tourism, the All-China Federation of Trade Unions, and the Ministry of Housing and Urban–Rural Development. Based on the nature of the issuing entities, these entities can be categorized into three tiers: The first tier is the National People’s Congress (Standing Committee), whose policy documents hold the highest policy authority; The second tier consists of the Central Committee of the Communist Party of China and the State Council, whose policy documents, whether issued separately or jointly, have legal authority second only to laws; The third tier includes policy-issuing entities such as departments under the Central Committee of the Communist Party of China, constituent departments of the State Council, directly affiliated institutions and public institutions, and mass organizations at the central level. Although entities in this tier have less policy influence than the first two tiers, they account for the largest proportion of the total policy volume.

#### 3.2.2 Individual policy entities

In terms of policy documents issued by individual policy-making entities, only three entities have independently issued policy documents related to National Fitness: the General Administration of Sport of China, the State Council, and the National People’s Congress. These entities have collectively issued 85 policy documents, accounting for 58% of all policy texts. Among these, the General Administration of Sport of China has independently issued 56 policy documents, representing 39% of the total, with over 60% of the independent policy documents originating from this entity. It is evident that policies on National Fitness are primarily formulated and issued by individual policy-making entities. Additionally, as the government department responsible for sports development, the General Administration of Sport of China plays a significant role in the formulation of National Fitness policies. Although other functional departments and units of the Central Committee of the Communist Party of China and the State Council have not independently formulated National Fitness policies, the State Council and the National People’s Congress possess high authority, and the policy documents they formulate not only reflect the reform direction of a particular field but also serve as important references for local governments in policy formulation and implementation. The 25 National Fitness policies independently formulated by the State Council have enhanced the overall effectiveness of China’s National Fitness policies, demonstrated the central government’s determination to promote National Fitness to the entire society, and further strengthened the top-level design and policy support for National Fitness.

#### 3.2.3 Joint policy entities

Multi-stakeholder collaborative governance is an inevitable choice and an intrinsic requirement for advancing the modernization of governance in the field of National Fitness. Joint policy issuance by multiple stakeholders is a hallmark feature of collaborative governance, as it can effectively prevent conflicts and coordination issues arising from different stakeholders acting independently, thereby enhancing the effectiveness of policies. Additionally, the form of jointly policies facilitates the proactive and proactive involvement of all stakeholders in addressing issues related to National Fitness. As of the end of 2021, Statistics show that there are currently 60 policy documents issued by joint entities in China’s National Fitness sector, accounting for 41% of the total number of documents (Table 6). Among the policy entities issuing joint documents, the majority are joint initiatives involving two departments, with a total of 28 documents issued, accounting for 46.7% of the total number of joint documents. The document titled “Opinions on Further Strengthening the Development of older adult(s) Culture,” issued by the National Committee on Aging as the leading entity, is the normative document with the most joint entities among all National Fitness policies, involving 16 policy entities such as the Ministry of Education, the Ministry of Civil Affairs, and the Ministry of Finance. The content not only emphasizes the necessity of providing sports and fitness services for the older adult(s) but also provides specific policy arrangements regarding the construction of sports infrastructure for the older adult(s) and the organization of sports and fitness activities. This indicates that promoting National Fitness is not solely the responsibility of the sports department but requires other departments to actively integrate National Fitness into their policy frameworks with an open mindset.

**Table 6:** Number of jointly issued National Fitness policy documents in China.

Number of collaborating departments	Number of documents issued	Percentage (%)
2	28	46.7
3	5	8.2
4	6	10.0
5	2	3.3
6	3	5.0
7	4	6.7
8	3	5.0
10	1	1.7
11	3	5.0
12	1	1.7
14	2	3.3
15	1	1.7
16	1	1.7
Total	60	100

From the perspective of the composition of entities involved in joint policy issuance, the General Administration of Sport of China is the entity with the highest number of participations, having participated in 55 instances, accounting for over 90% of all joint policy issuances. This reflects the sports authorities’ emphasis on fulfilling their responsibilities in providing public fitness services and their proactive response to the public’s actual needs for such services through multi-departmental collaboration. The Ministry of Education follows closely behind with 21 instances of joint policy issuance, accounting for 35% of the total. Other departments that have been actively involved in the formulation of National Fitness policies include the Ministry of Education (21 times), the National Health Commission (16 times), the Ministry of Civil Affairs (15 times), the Ministry of Finance (15 times), the National Development and Reform Commission (14 times), the Ministry of Culture and Tourism (12 times), the All-China Federation of Trade Unions (10 times), and the Ministry of Housing and Urban–Rural Development (10 times). The Ministry of Justice, National Bureau of Statistics, Ministry of Transport, and 15 other departments had limited involvement, with only one policy document issued. Further statistics show that, as shown in Table 7, 15 policy-making entities served as lead departments in issuing National Fitness policy documents. Among them, the General Administration of Sport of China led the development of the largest number of policy documents, totaling 30. The Central Committee of the Communist Party of China, the National Development and Reform Commission, the Ministry of Education, the General Office of the State Council, the National Health Commission, and the China Disabled Persons’ Federation, among others, issued at least two policy documents each. The Ministry of Public Security, the Ministry of Agriculture and Rural Affairs, and five other departments led the issuance of one policy document each. Leading departments bear primary responsibility for joint policy issuance, overseeing implementation and driving policy advancement. Non-leading departments primarily assist the lead departments in their work, assuming coordination and support roles. This indicates that the Ministry of Education, the Ministry of Finance, the National Development and Reform Commission, and the National Health Commission, among others, not only have a broad scope of participation in promoting National Fitness but also, as core issuing institutions, demonstrate a high level of enthusiasm and intensity in participating in National Fitness construction, playing a significant role in the formulation of China’s National Fitness policies.

**Table 7:** Number of National Fitness policy documents issued by leading departments in China.

Leading department	Number of documents issued
Ministry of Finance	3
National Development and Reform Commission	4
Ministry of Public Security	1
State Ethnic Affairs Commission	1
General Administration of Sport of China	30
General Office of the State Council	3
Ministry of Education	4
Ministry of Agriculture and Rural Affairs	1
National Committee on Ageing	1
Ministry of Human Resources and Social Security	1
National Health Commission	2
Ministry of Culture and Tourism	1
China Disabled Persons’ Federation	2
Communist Party of China Central Committee	5
Ministry of Housing and Urban–Rural Development	1
Total	60

From the perspective of joint policy document categories, notices and opinions constitute the primary types of policy documents, with a total of 37 such documents accounting for 61.7% of all joint policy documents. Notices and opinions are administrative regulations that possess authority but are not formal laws. They primarily emphasize administrative guidance (24). As such, joint policy documents overall exhibit weaker normative and mandatory characteristics, tending to assume the role of “facilitators” or “supporters” in promoting the development of National Fitness, manifested through cooperation and assistance rather than the use of non-mandatory policy measures to drive the development of National Fitness.

Based on the degree of joint issuance, government departments can be categorized into three types of joint issuance based on the number of departments involved: high-level joint issuance (2–3 departments), moderate-level joint issuance (4–6 departments), and low-level joint issuance (7 or more departments) (25). It can be observed that the degree of joint issuance for National Fitness policies is relatively high, with 33 policy documents classified as high-level joint issuance, accounting for 55% of the total. The higher the level of joint issuance, the easier it is to implement policies, and the better the outcomes achieved. Therefore, overall, joint policy documents related to National Fitness have a solid foundation for implementation. However, a significant portion of low-level joint issuance documents (16 documents) require attention. These policies involve a large number of departments, which face challenges such as unclear functional roles and slow information communication, significantly reducing the operability and enforceability of the policy texts.

#### 3.2.4 Empirical analysis of policy-making entity cooperation networks

Collaborative cooperation among policy-making entities is essential for the efficient development of National Fitness programs. A comprehensive understanding of the structural characteristics of the collaborative network among National Fitness policy-making entities and an exploration of the interactive relationships among different policy-making entities can not only identify the degree of connection among policy-making entities, providing further reference for strengthening collaborative cooperation among them, but also, by understanding the structural positions of each entity within the collaborative network, help identify reasonable and effective solutions to enhance the implementation efficiency of China’s National Fitness policies.

**Figure 4:**
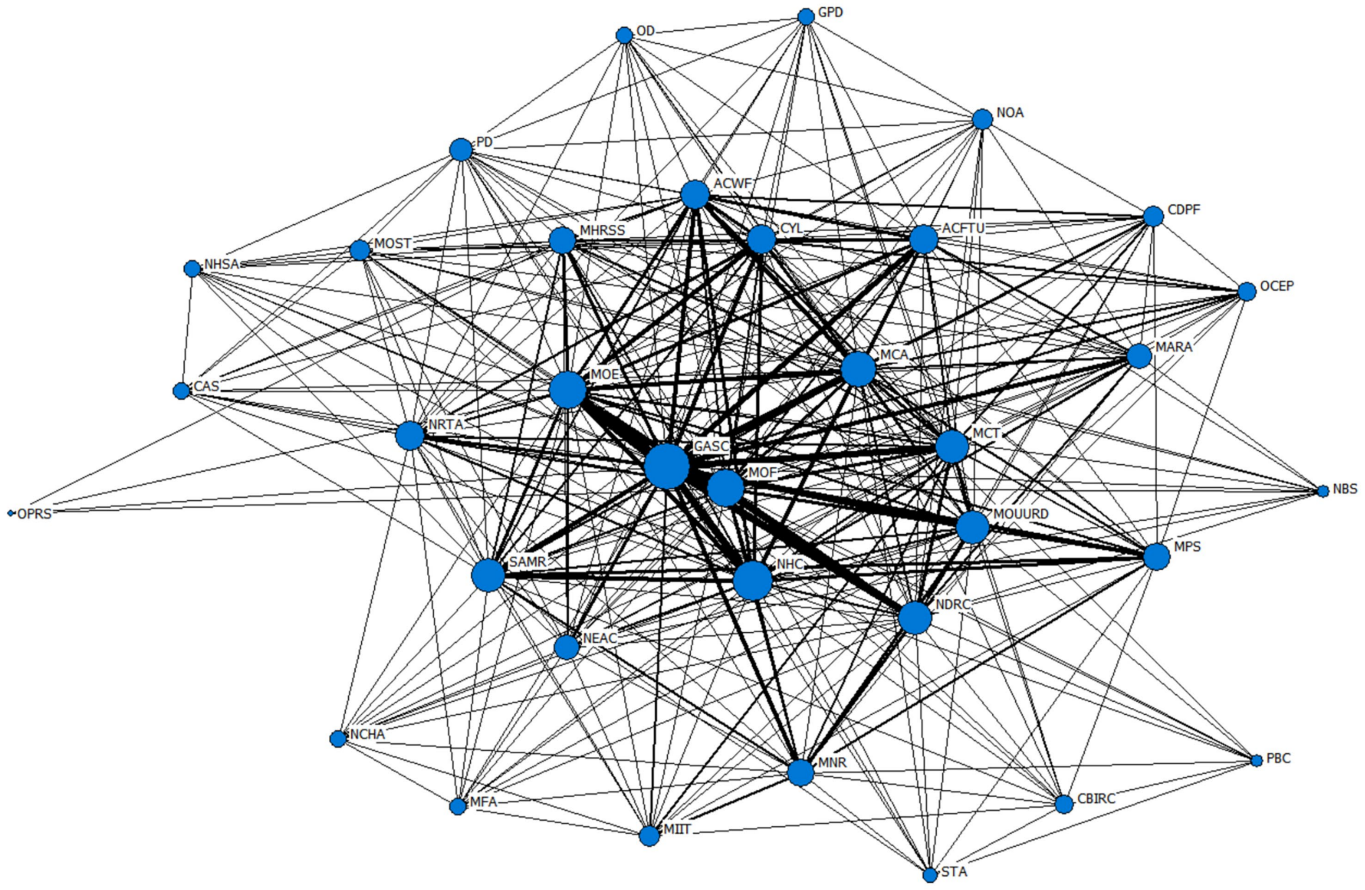
Network diagram of cooperation between policy-making bodies for National Fitness policies in China.

Based on the 60 joint policy documents on National Fitness obtained from the aforementioned research, a statistical analysis was conducted on the collaborative relationships among the policy entities. In terms of relationship assignment, if two policy entities have jointly issued documents, the relationship between them is assigned a value of 1; otherwise, it is assigned a value of 0. Based on this, the joint issuing entities of the National Fitness policy are obtained. Co-occurrence matrix. Subsequently, the UCINET 6.560 software was used to calculate the cooperation network structure indicators, and the Netdraw software was used to draw the cooperation network relationship diagram of policy entities in China, as shown in Figure 4.

In the network diagram, nodes represent policy-issuing entities, and the lines connecting nodes indicate collaborative policy-issuing relationships between entities. Larger node sizes indicate higher frequencies of collaborative policy issuance, while thicker lines between nodes signify closer cooperative relationships between entities. As such, on one hand, the General Administration of Sport of China, the National Health Commission, the Ministry of Education, the Ministry of Finance, the Ministry of Civil Affairs, the National Development and Reform Commission, the Ministry of Culture and Tourism, the Ministry of Housing and Urban–Rural Development, and the State Administration for Market Regulation occupy central positions in the cooperative network of policy-issuing entities for National Fitness. These entities have larger node sizes and higher connection densities both among themselves and with other policy-issuing entities, thereby holding significant authority within the cooperative network. The State Council and the Central Committee of the Communist Party of China, as the highest authoritative bodies of the state, are the superior departments of other policy-making entities. Although they do not jointly issue documents with other departments and are located at the periphery of the policy-making entity network, their policy status is high, determining the direction of action for other policy-making entities. On the other hand, the cooperation network density of China’s National Fitness policy-making entities is 0.405, a relatively weak value, while the network centrality of the cooperation network is 57.6%, a relatively high value. This indicates that the current cooperation network of China’s National Fitness policy-making entities tends toward a central-peripheral network structure (26), with weak connectivity in the overall policy network structure and an unbalanced distribution of network space. Only some policy-making entities have close connections, and the network structure lacks stability.

“Centrality” analysis is an important tool for measuring the “power” that actors hold within a network. In other words, to understand the status and influence of policy-making entities, it is necessary to measure the centrality of policy-making actors, specifically by calculating three types of centrality metrics: degree centrality, intermediate centrality, and proximity centrality (as shown in Table 8). The higher a node’s degree centrality, the more central its position within the network, and the stronger its influence within the collaborative network. The betweenness centrality of a node indicates to what extent it is located between two other nodes, reflecting its control capacity within the network. The higher the proximity centrality of a node, the farther it is from the central node. From this perspective, General Administration of Sport of China has the highest degree centrality and intermediate centrality, indicating that it plays a prominent role in the policy cooperation network for the promotion of physical fitness in China. The National Health Commission, Ministry of Education, Ministry of Finance, and Ministry of Civil Affairs are also important nodes in the network, having issued a series of policy documents to actively promote the development of physical fitness and playing a significant role in the policy cooperation network.

**Table 8:** Centrality indicators of collaborative actors in China’s National Fitness policies.

Rank	Node	Degree centrality	Node	Betweenness centrality	Node	Closeness centrality
1	General Administration of Sport of China	43	General Administration of Sport of China	125.43	State Council, CPC Central Committee	2025
2	National Health Commission	37	National Health Commission	50.99	All-China Sports Federation	175
3	Ministry of Education	35	Ministry of Education	48.55	State Commission Office for Public Sector Reform	174
4	Ministry of Finance	34	National Development and Reform Commission	42.22	Ministry of Water Resources, Agricultural Development Bank of China, Work Committee of Central and State Organs	172
5	Ministry of Civil Affairs	32	Ministry of Finance	36.37	People’s Bank of China	169

### 3.3 Policy themes

Theme strength reflects the relative weight of each theme within the entire policy text corpus, thereby indicating the current level of attention given to certain themes in China’s National Fitness policies. A higher theme strength indicates that the theme is a key focus of government attention. The formula used in this study to calculate the theme strength of National Fitness policies is:

$P_{k}+\frac{\sum_{i}^{N}\theta_{\mathrm{ki}}}{N}$

**Figure 5:**
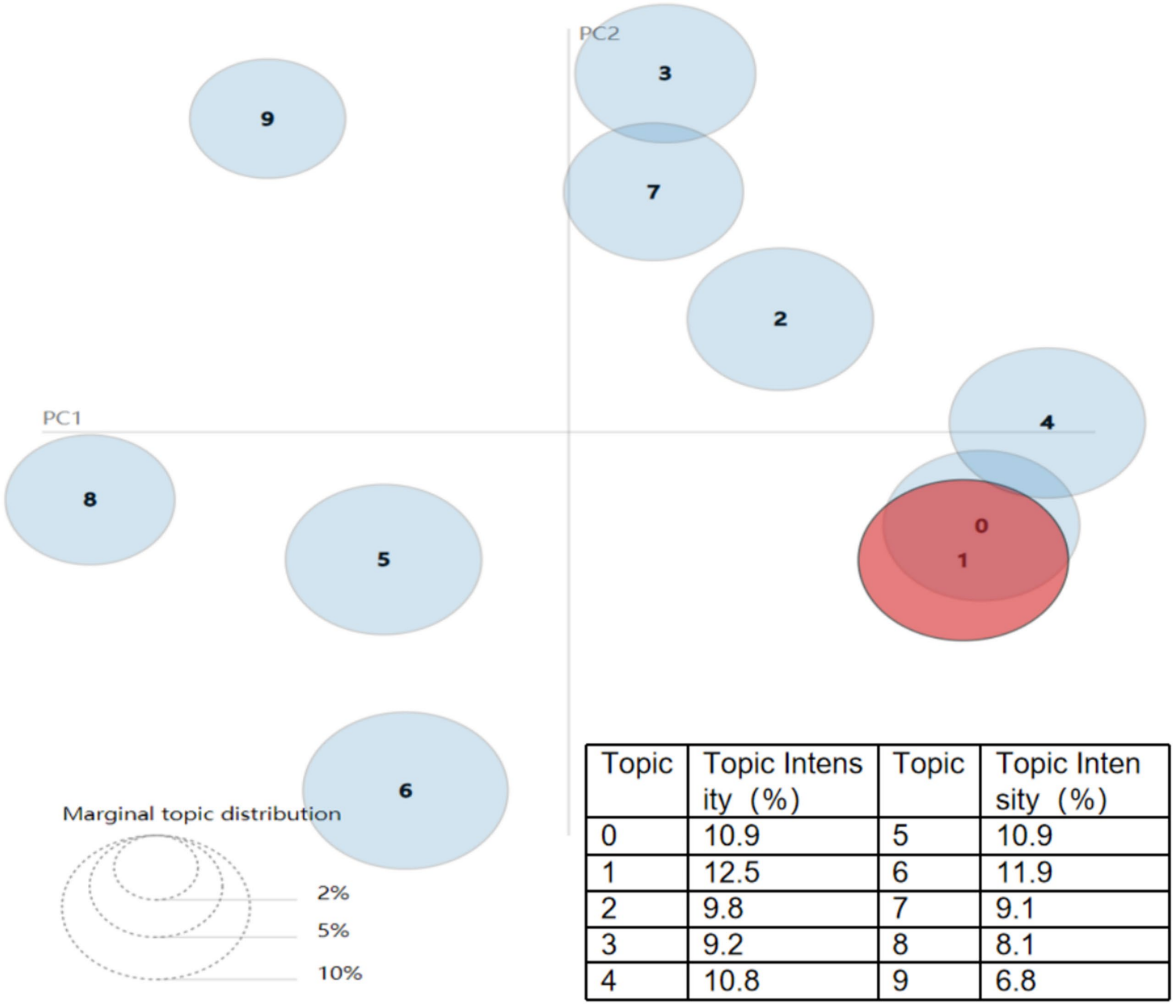
Results of LDA topic model analysis of China’s National Fitness policy texts.

Here, *P_k_* represents the strength of the kth theme, *N* denotes the number of documents, and *θ_ki_* indicates the probability of the kth theme appearing in the *i*th document (27). The results show that the distribution of theme strengths across China’s National Fitness policies is illustrated in Figure 5. In the topic visualization diagram generated by the py LDAvis library, each circle represents a topic, with larger circles indicating a higher number of texts associated with that topic. The distance between circles reflects the degree of connection between policy text topics, with closer distances indicating stronger connections and higher similarity. As such, topics three and seven, as well as topics zero, one, and four, exhibit strong associations. Based on theme strength, Theme 1, Theme 6, Theme 5, and Theme 0 rank in the top three, with respective percentages of 12.5, 11.9, and 10.9%. Theme 7, Theme 3, and Theme 8 rank in the bottom three, with respective percentages of 9.2, 9.1, and 8.1%. Therefore, from the perspective of theme strength, the similarity between policy themes is not high, and the differences are not significant, reflecting the coordination and balance of China’s National Fitness policy texts.

After obtaining the terms and their relevance from the LDA analysis of the thematic content of China’s National Fitness policies, and based on the terms with higher frequencies under each theme, while referencing the National Fitness policy document repository, the explanatory names for different policy themes can be determined (Table 9), namely: fitness for key populations, fitness and leisure industry, school sports activities, National Fitness events and activities, National Fitness sports programs, National Fitness “streamlining administration and delegating power” reform, National Fitness facilities and venues, National Fitness guidance, National Fitness funding, and National Fitness organizations and culture. Based on the content characteristics of the themes, the themes of China’s National Fitness policies can be further categorized into three prominent issues: fitness for key populations, the National Fitness public service system, and National Fitness governance.

**Table 9:** Keywords and relevance of thematic content in China’s National Fitness policies.

Related theme	0 Key populations		1 Fitness & leisure industry		2 School sports activities		3 National Fitness events and activities		4 National Fitness sports programs	
	Term	Relevance	Term	Relevance	Term	Relevance	Term	Relevance	Term	Relevance
National Fitness policy theme	Older adult(s)	0.03509	Sports Industry	0.05960	Schools	0.07108	Events	0.11317	Football	0.11719
	Fitness	0.03495	Fitness and Leisure	0.04165	Students	0.07004	Athletes	0.06066	Winter Sports	0.08491
	Rural Areas	0.03080	Services	0.0360	School Sports	0.05122	Competitions	0.04834	Martial Arts	0.04795
	Farmers	0.02826	Training	0.02639	Sports activities	0.03031	Sports meets	0.03473	Society	0.02652
	Disabled persons	0.02638	Enterprises	0.02170	Physical fitness	0.02852	Participation	0.01424	Qigong	0.01902
	Services	0.02585	Industry	0.01982	Youth Sports	0.02837	Association	0.01360	City	0.01902
	Sports Facilities	0.02183	Mechanism	0.01748	Education	0.02703	Coaches	0.01296	Clubs	0.01352
	Mass Participation	0.01835	Tourism	0.016192	Standards	0.02329	Doping	0.01184	Schools	0.01312
	Traditional Sports Events	0.01687	Event Activities	0.01560	Training	0.01941	Prevention and Control	0.01056	Mechanism	0.01272
	Society	0.01567	Market	0.01501	Extracurricular	0.01538	Referee	0.01056	Football Field	0.01245

Related topics	5 “Streamlining administration and delegating power” reform		6 National Fitness facilities		7 National Fitness guidance		8 National Fitness funding guarantees		9 National Fitness organization and culture	
	Term	Relevance	Term	Relevance	Term	Relevance	Term	Relevance	Term	Relevance
National Fitness policy theme	Management	0.08535	Sports facilities	0.09842	Social sports instructors	0.05685	Funding	0.05794	Clubs	0.03878
	Services	0.06145	Sports Venues	0.06381	Coaches	0.02794	Management	0.05611	Associations	0.03374
	Standards	0.03498	Equipment	0.04493	Society	0.02584	Sports Administration Department	0.04071	Sports Culture	0.03330
	Supervision	0.03282	Facilities	0.03989	Training	0.02439	Public Welfare Fund	0.03410	Family	0.02169
	Regulations	0.03052	Management	0.03134	Volunteerism	0.02002	Funding	0.02402	Institution	0.02147
	Systems	0.02741	Land Use	0.02429	Training	0.01922	Sports Lottery	0.01962	Children	0.016215
	Society	0.02417	Operations	0.02190	Mass Fitness Volunteer Services	0.01776	Central Government	0.01540	Management	0.01490
	Sports Administrative Department	0.01553	Park	0.01799	Team	0.01615	Publicity	0.01503	Social Sports	0.01402
	Vocational Skills	0.01526	Operations	0.01447	Associations	0.01550	Supervision	0.01485	Guidance	0.01314
	Information	0.01283	Risk	0.01296	Services	0.01486	Subsidies	0.01448	Lottery	0.01205

(1) Fitness for key populations. China’s National Fitness policies focus on key populations, including adolescents, the older adult(s), farmers, and people with disabilities. The government has implemented a series of measures to promote youth health, shifting from the previous “physical development” concept to a “health first” approach, guiding youth sports and fitness through reforms in physical education and sports curricula. Cultural development in youth sports, such as the promotion of campus football and winter sports, has also been prioritized. Standards for student physical fitness have been established, along with policy support for youth sports funding, sports club development, facility access, and time allocation for sports activities. The physical fitness status of youth has become a key component in provincial-level assessments of myopia prevention for children and adolescents.

Farmers, central to rural revitalization, are a key focus for fitness policies, with the government emphasizing the improvement of the rural sports public service system. Through initiatives like the Farmers’ Sports and Fitness Program, the establishment of township cultural stations, and the promotion of agriculture-sports integration, fitness has been integrated into farmers’ daily lives. The issue of farmers’ fitness is frequently addressed in key policy documents, such as Central Document No. 1.

For people with disabilities, the government has made significant efforts to ensure participation in sports activities, leveraging the development of disability sports events to raise awareness. The integration of disability sports into the broader national development framework has been promoted, with a focus on rehabilitation and fitness services tailored to their specific needs, including accessibility standards, sports instructor training, and specialized events like Disability Fitness Week.

Regarding older adult(s) sports policies, the government has worked to improve the foundational conditions necessary for older adult(s) fitness activities, including facility construction, funding management, professional training, and sports organization development (28). Specific measures have also been implemented to address the challenges older adult(s) individuals face in using smart technologies in public sports services. Non-medical health interventions for the older adult(s) have been promoted, ensuring the integration of fitness and health for aging populations.

(2) Public Service System for National Fitness. The public service system for National Fitness is a crucial cornerstone for promoting National Fitness in China. This topic encompasses Theme 3 (National Fitness Events and Activities), Theme 4 (National Fitness Sports Programs), Theme 6 (National Fitness Facilities), Theme 7 (National Fitness Guidance), Theme 8 (National Fitness Funding), and Theme 9 (National Fitness Organizations and Culture). A review of these themes and policy documents reveals that, in terms of events, the approval process for commercial and mass sports events has been abolished, and a standardized system for organizing National Fitness events has been established, focusing on safety, service guidance, and organizational support. Regarding sports programs, efforts are focused on promoting the healthy development of football, winter sports, fitness qigong, and other projects, with specialized policies to ensure their success. For facilities, solutions to issues such as land acquisition, facility accessibility, and funding shortages have been addressed through the establishment of guidelines and supply standards. In the realm of National Fitness guidance, emphasis is placed on developing the social sports instructor system, part-time coach positions, and National Fitness volunteer services, thereby improving the quality of fitness guidance. Regarding funding, the government has clarified its responsibility for fiscal subsidies and emphasizes increased investment from the Sports Lottery Public Welfare Fund to support the development of National Fitness. In terms of organizations and culture, efforts are being made to decouple sports associations from administrative departments and to foster the development of sports social organizations at all levels. Moreover, there is strong support for the construction of Chinese sports spirit, traditional sports culture, sports project culture, and sports cultural products, aiming to create a diverse and multifaceted sports culture.
(3) Modernization of governance capabilities for National Fitness. The modernization of governance capabilities for National Fitness is a vital guarantee for ensuring the healthy and orderly development of National Fitness in China. Themes 5 and 1 highlight that the reform of government functions and the development of the fitness and leisure industry are key focuses of the government’s efforts to modernize National Fitness governance. The policy measures include the establishment of an inter-ministerial coordination mechanism for National Fitness, which has effectively promoted resource integration and collaboration, ensuring the implementation of National Fitness initiatives. By 2018, 28 provinces (regions, municipalities), 328 cities, and 1,723 counties had established government-led leadership and coordination mechanisms for National Fitness (29). Market mechanisms have been further strengthened, with specific policies such as the decoupling of sports industry associations, the establishment of a regulatory affairs directory list for the sports system, a sports market blacklist, and the cancellation of approval requirements for mass sports events, all of which have provided more resources and opportunities for market entities. Innovative operational mechanisms have been introduced, including the development and promotion of technical grading standards for amateur athletes. The implementation of routine and innovative actions, such as the “Three-Level Joint Creation for National Fitness,” “Sports Consumption City Pilot Programs,” and “Three Inclusions for National Fitness,” has effectively stimulated participation from individuals and organizations. The evaluation mechanism for National Fitness has been enhanced, with clear evaluation standards and procedures, involving multi-party assessments by the General Administration of Sport, provincial sports departments, and third-party organizations, ensuring the scientific rigor of the evaluation process.

### 3.4 Policy tools

Ultimately, the usage of policy tool types in China’s National Fitness policy texts was statistically analyzed (as shown in Table 10). The analysis reveals the following characteristics:

**Table 10:** Statistical table on the use of policy tool types in China’s National Fitness policies.

Tool type	Tool name	Number (times)	Tool type proportion (%)
Voluntary policy tools	Voluntary Organizations	55	3.9
	Family and Community	18	1.3
	Market	64	4.6
	Total	137	9.8
Mandatory policy tools	Regulation	266	18.9
	Direct Provision	290	20.7
	Command and Authority Tools	405	28.8
	Total	961	68.4
Hybrid policy tools	Information and Advice	189	13.5
	Subsidies	34	2.4
	Contracts	30	2.1
	Incentive-based tools	53	3.8
Total		306	21.8
Grand total		1,404	100

(1) Insufficient use of voluntary policy tools. Overall, in the selection structure of policy tools in China’s National Fitness policy texts, voluntary policy tools were selected extremely rarely, with only 137 instances, accounting for 9.8% of the total number of tool types used in the sample. Compared to mandatory and hybrid policy tools, voluntary policy tools are significantly underutilized. Specifically, the three measures—“voluntary organizations,” “family and community,” and “market”—account for very small proportions in the use of policy tools in National Fitness policy texts, at 3.9, 1.3, and 4.6%, respectively. As a type of policy tool that involves minimal or no government intervention, voluntary policy tools have the advantage of leveraging the roles of society and the market, reducing government costs, and, due to their foundation on free choice, facilitating the implementation and advancement of policies. Therefore, the underutilization of voluntary policy tools can lead to increased implementation costs for National Fitness policies and hinder their execution.
(2) Excessive use of coercive policy tools. Coercive policy tools refer to strong intervention measures taken by the government to solve specific social problems by restricting the behavior of certain target groups and individuals. As shown by the results, mandatory policy tools appear with high frequency in the policy tool selection structure of China’s National Fitness policy texts, totaling 961 instances, accounting for 68.4% of the total—nearly 10 times that of voluntary policy tools. Among these, regulatory tools, direct provision tools, and authoritative tools account for 18.9, 20.7, and 28.8%, respectively. From a holistic perspective, the use of coercive policy tools in the implementation of China’s National Fitness policy texts is excessive. In all 145 policy documents, nearly all emphasize the use of standards, rules, supervision, and penalties to ensure the implementation of National Fitness policy texts. Mandatory policy tools have the advantages of being direct and swift, effectively ensuring the implementation of National Fitness policies and to some extent resolving related policy issues. However, excessive reliance on such tools can stifle the flexibility of government departments in policy implementation, often leading to waste or inefficiency in National Fitness public services. This not only hinders local governments from developing tailored solutions but also risks concentrating excessive administrative power in the government, thereby obstructing social groups’ participation and implementation of policies.
(3) The selection structure of hybrid policy tools is unreasonable. Hybrid policy tools combine the dual characteristics of government intervention and autonomy. On the one hand, they require the government to intervene appropriately, and on the other hand, they encourage non-governmental organizations and institutions to participate in the decision-making process. In the policy tool types of China’s National Fitness policy text, hybrid tools account for 21.8% of the total, which is a moderate proportion. However, within their microstructure, the allocation of hybrid policy tool types is unreasonable. Among these, “information and persuasion” appears 189 times, accounting for 13.5%, ‘subsidies’ appear 34 times, accounting for 2.5%, “incentive-based tools” appear 53 times, accounting for 3.8%, while “contracts” are the lowest, appearing 30 times, accounting for only 2.1%. In the policy texts on National Fitness, hybrid policy tools primarily rely on “information and persuasion” measures. Based on the coding analysis, among “information and persuasion” measures, the most frequently used is “encouragement and advocacy.” However, policy statements emphasizing terms like “encourage,” “advocate,” and “require” have limited impact and implementation effectiveness, which can easily undermine the policy’s execution outcomes.

## 4 Conclusion

The findings reveal that, overall, China’s National Fitness policy supply exhibits the following characteristics:

First, there is a clear trend toward concentration in the distribution of policy numbers, and the timeliness of policies still needs to be further improved. Most of the currently effective National Fitness policy texts in China were issued and implemented after the country entered the new era of socialism with Chinese characteristics, aligning with socio-economic development and possessing a certain degree of timeliness. However, some policy documents remain outdated and lag behind, impacting the achievement of National Fitness policy objectives, which requires our attention and emphasis.

Second, the effectiveness of policy texts has transitioned from fluctuating to stabilizing, with overall strong applicability. Following the 18th National Congress of the Communist Party of China, as government attention in the field of National Fitness increased, a series of institutional documents were issued and implemented, leading to the effectiveness of National Fitness policy texts gradually entering a stable state. Policies increasingly exhibit a distribution pattern characterized by “strong regulatory control and high subject-relatedness,” with strong applicability. However, it is important to emphasize that, as China has not yet established specialized legislation for National Fitness, the existing laws and regulations, including the National Fitness Regulation, suffer from issues such as low legal standing and insufficiently detailed provisions, severely constraining the implementation effectiveness of National Fitness policies (30).

Third, while there are diverse policy-making entities, the stability of the policy network structure remains to be improved. In addition to the General Administration of Sport of China, the national authority responsible for sports affairs, 46 other entities, including the Ministry of Education, the Ministry of Finance, and the National Ethnic Affairs Commission, have participated to varying degrees in the formulation of National Fitness policies. However, the results of Social Network Analysis (SNA) indicate that the governance structure is highly concentrated in the General Administration of Sport of China. This “single-center” structure has become a structural barrier to the integration of sports and healthcare (“sports–medicine integration”). It is crucial that the current fragmented situation undermines the core goal of Universal Health Coverage (UHC)—which is to provide prevention-oriented, comprehensive health services. At present, the policy framework for National Fitness overly relies on sports administrative departments, with insufficient deep collaboration with health and medical authorities. As a result, the potential of National Fitness as a high-cost-effective tool for chronic disease prevention has not been fully realized, while chronic disease prevention is a key pillar for China’s sustainable UHC. This structural defect exacerbates the disconnection between policy-making across different government departments, leading to unclear responsibilities during policy implementation and restricting the efficiency of non-medical interventions at the societal level.

Fourth, policy themes focus on three key issues: fitness for key populations, the National Fitness public service system, and National Fitness governance. Using LDA topic clustering, the text of China’s National Fitness policies can be divided into ten thematic categories. The top five themes by strength are fitness and leisure industry, National Fitness facilities, fitness for key populations, “streamlining administration and delegating power” reform, and National Fitness sports events; the bottom five themes are school sports activities, National Fitness competitions, National Fitness guidance, National Fitness funding, and National Fitness organizations and culture. Based on this, the same themes were further merged to identify three prominent issues: fitness for key populations, the public service system for National Fitness, and governance of National Fitness. Comparing the “Outline for Building a Sports Powerhouse” and the “National Fitness Plan (2021–2025),” it is evident that there are certain gaps in policy content, with insufficient attention given to two key issues: new models for promoting health through exercise and the smartification of National Fitness. This hinders the improvement of the quality and level of National Fitness development.

Fifth, policy tools are primarily coercive in nature, and there is room for optimization in the tool structure. Based on Ramesh’s policy tool theory, MAXQDA Analytics Pro 2022 qualitative analysis software was used to analyze the use of tools in China’s National Fitness policy texts, revealing an imbalance in policy tool application. Specifically, there is excessive reliance on coercive policy tools such as regulation and direct provision, while voluntary policy tools are underutilized. This imbalance presents a significant challenge, as it limits the mobilization of social forces, which is crucial for achieving the goal of Universal Health Coverage (UHC). UHC cannot be achieved solely through government coercive measures; it also requires voluntary public participation and active involvement from market entities to create a supportive health environment. The current lack of voluntary policy tools hinders the formation of a bottom-up health promotion mechanism, thereby slowing down and limiting the speed and scope of achieving the policy objectives.

Although this study conducted a comprehensive literature search and inclusion, the strict standards for public accessibility may have excluded non-public documents such as internal administrative notices. This potential limitation may affect the completeness of the review.

## 5 Policy recommendations

### 5.1 Strengthen the timeliness of policy supply

The timeliness of policy implementation directly influences its effectiveness. As of the end of 2021, China’s National Fitness policies suffer from a lag in supply, which has hindered progress in promoting National Fitness, making it difficult to advance certain initiatives. For instance, with the launch of the Healthy China strategy, the integration of National Fitness and public health requires effective policy guidance. However, the National Fitness Regulations, as the primary legal framework for promoting public health, only contain three provisions related to health protection (31), which are insufficient and do not actively promote the integration of National Fitness with public health (32).

To ensure the timely supply of National Fitness policies in China, it is essential to adhere to a dual approach combining top-level design with an experimental, trial-and-error method. Top-level design serves as the comprehensive plan for National Fitness development, involving multiple sectors, including the Ministry of Education, the Ministry of Finance, and the Ministry of Public Security. Strengthening this approach ensures a cohesive, comprehensive policy supply, thereby avoiding fragmentation and ensuring timeliness. Furthermore, “feeling our way forward” is a core method derived from China’s socio-economic development, requiring careful, progressive policy testing rather than blind advancement. The central government should conduct top-down policy experiments to address long-standing challenges and encourage local governments to experiment within the framework of top-level design, fostering innovation in National Fitness policy.

### 5.2 Promote the coordinated design of policy texts

The quality of National Fitness policy texts is crucial for their effective implementation (33). Based on the analysis, it is vital to enhance coordination between policy-making agencies, policy types, and policy tools to improve the quality of National Fitness policy texts in China. Specifically,

First, strengthen coordination between the sports departments and other key departments responsible for resources such as land, talent, taxation, and publicity. Enhancing interaction between these departments will help establish a long-term mechanism for the coordinated design of National Fitness policy texts. Furthermore, the implementation of the “National Fitness Integrated into All Policies” initiative should be advanced by issuing detailed guidelines for integrating National Fitness into transportation, environmental, agricultural, and food policies.

Second, focus on the coordination of policy text structures. Accelerate the development of laws and regulations related to public fitness, clarify the public’s right to engage in fitness activities, and improve penalties for unlawful actions that infringe on National Fitness. Add macro-level policies to address current challenges and improve the operability of policy texts.

Third, emphasize the synergistic use of three types of policy tools: voluntary, mandatory, and hybrid. Increase the use of voluntary policy tools to highlight the role of sports organizations, families, communities, and the market in promoting fitness activities. Reduce the use of mandatory tools while innovating policy measures, such as using modern information technology to improve the formulation, implementation, and evaluation of National Fitness policies. Efforts should also be made to avoid the overuse of coercive measures and to ensure clearer expressions in policies related to subsidies, preferential taxes, and incentives.

### 5.3 Align policy focus with Universal Health Coverage (UHC) objectives

To fully unlock the potential value of National Fitness in advancing Universal Health Coverage (UHC), it is crucial for policymakers to strengthen their focus and strategic planning on two key issues: the intelligent development of National Fitness and the promotion of a new “sports-health integration” model for exercise-based health promotion. On one hand, the deep integration of smart technologies with National Fitness is not merely a technological upgrade; it is a critical pathway to expanding the coverage of health services and alleviating the economic burden of healthcare. On the other hand, the implementation and promotion of the “sports-health integration” model can fully harness the unique advantages of sports in disease prevention and health promotion, making it indispensable for advancing preventive health measures within the UHC framework. However, current policy support in these areas remains limited and requires more targeted attention to unlock their full potential.

The integration of smart technology has become a key means to enhance the precision, convenience, and efficiency of National Fitness services, as well as to foster the development of new fitness industries and promote the sharing of resources. The “integration of fitness and health” model is critical for advancing the deep integration of National Fitness and public health, and for improving preventive health measures. However, policy support for these areas remains limited and deserves more attention.

To promote the intelligent development of National Fitness as a support system for UHC, policies should prioritize the following: (1) Enhance the intelligence of fitness facilities by leveraging IoT, cloud computing, and big data to upgrade sports venues and improve the management of fitness facilities. (2) Build a public information service platform for National Fitness, encouraging the participation of market and social forces to integrate data on facilities, events, and fitness knowledge. (3) Establish a comprehensive database for National Fitness and develop real-time monitoring and integrated management systems using 5G and AI technology. (4) Set up smart fitness demonstration zones and pilot projects, ensuring technical standards and information security, while promoting standardized construction and management of fitness facilities.

For the “fitness-health integration” model, which serves as the operational mechanism for connecting National Fitness with UHC, the following measures should be implemented: (1) Define the responsibilities of sports and related departments, integrate fitness into all policies, and improve the efficiency of National Fitness initiatives. (2) Develop scientific fitness programs based on the National Fitness Guidelines and promote them through public education, welfare programs, and sporting events. (3) Build a scientific fitness information service platform to encourage intelligent participation in National Fitness activities. (4) Strengthen the planning of sports-medicine integration demonstration zones, cultivate interdisciplinary talent, and improve standards and funding support. (5) Establish a community-based proactive health service platform to monitor residents’ health, provide personalized fitness programs, and target interventions for chronic diseases, obesity, and the older adult(s).

Ultimately, the optimization of China’s National Fitness policies should be viewed as an indispensable part of the national Universal Health Coverage (UHC) roadmap. By addressing the issues of policy lag, structural fragmentation, and imbalanced tools identified in this study, China can transform National Fitness from an independent sports development initiative into a foundational pillar of its public health system. This transformation is crucial for shifting the focus of UHC from “disease treatment” to “active health promotion,” ensuring that health services are not only accessible to all but also provide proactive preventive care for the entire population.

## Data Availability

The original contributions presented in the study are included in the article/Supplementary material, further inquiries can be directed to the corresponding author.

## Footnotes

1
https://www.pkulaw.com/
